# Surface nanoengineering technology for the removal of sulfur compounds associated with negative attributes in wines

**DOI:** 10.1038/s41538-023-00180-8

**Published:** 2023-02-08

**Authors:** Agnieszka M. Mierczynska-Vasilev, Allie C. Kulcsar, Panthihage Ruvini L. Dabare, Krasimir A. Vasilev, Marlize Z. Bekker

**Affiliations:** 1 The Australian Wine Research Institute, Waite Precinct, Hartley Grove cnr Paratoo Road, Urrbrae, Adelaide, SA 5064 Australia; 2 College of Medicine and Public Health, Flinders University, Sturt Road, Bedford Park, SA 5042 Australia

**Keywords:** Agriculture, Nanoscale materials

## Abstract

Volatile sulfur compounds (VSCs), such as hydrogen sulfide, methanethiol, and ethanethiol, are associated with ‘reductive’ aromas in wine and contribute to approximately 30% of all wine faults. These compounds can have a significant impact on wine aroma and perceived quality, and subsequently, consumer preference. In this communication, we report a method for the removal of VSC compounds based on nanoengineered surfaces that incorporate immobilized gold nanoparticles.

Volatile sulfur compounds (VSCs) can significantly impact the perceived quality and viability of wine production. While some VSCs positively contribute to fruity characters, others are associated with the undesired ‘reductive’ aromas (e.g., rotten egg, cabbage, burnt rubber, putrefaction, sulfurous). These characters in finished wine are considered winemaking faults and account for up to 30%^1^ of all faults detected in commercial wines. The prevention and management of ‘reductive’ aromas are of major importance to wine producers, especially considering that ‘reductive’ faults are not isolated to only a certain segment of wine producers but negatively impact both red and white wines, large- and small-scale producers. The main method for managing ‘reductive’ aromas is copper fining. The legal limit for residual copper in wine is 1.0 mg/L in the United States and the European Union due to health considerations and the negative impacts of copper on wine’s organoleptic properties. It is known that copper fining can be associated with increased oxidation, loss of sulfur dioxide, removal of desirable fruity, citrus, and tropical aromas^2^ and even might promote the formation of undesirable VSCs post-bottling^3,4^. Adopting a sustainable, non-toxic alternative to copper fining would therefore have the potential to provide beneficial environmental and economic impacts.

Herein, we present a new and facile method to eliminate key VSCs compounds from wines, combining chemical and structural surface modification. The technology is based on applying a thin plasma polymer coating to a surface and then immobilizing gold nanoparticles on that surface. We hypothesize that using gold nanoparticles would allow for the removal of VSCs from wine by creating gold-sulfur bonds since it is known that sulfhydryls bind strongly to gold surfaces. Our approach is shown in Fig. 1. We chose gold nanoparticles because they can be easily synthesized in a controlled manner and are chemically stable in the size range used in this study^5^. Supplementary Figs. 1 and 2 show the physicochemical properties and SEM examination of the nanoengineered surfaces. A key benefit of our approach is that it is an easily deployable and retrievable processing platform, making it a one-step process (a surface is added directly to the wine and then removed after a certain time period). The process can be repeated if necessary. In contrast, copper fining is a multi-step process. Copper ions bind to sulfur-containing compounds to form insoluble copper sulfides, which are then removed by cold settling or filtration. Recent work has highlighted the difficulties associated with the copper fining process and that up to 50% of the copper remains in wine post treatment^6^.

**Fig. 1:**
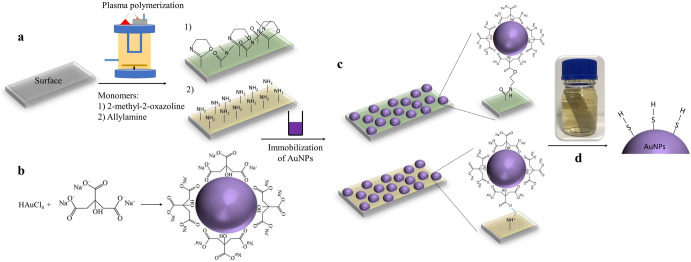
Schematic representation of the process of generation of nanoengineered surfaces and their utilization in wine. **a** Surfaces were coated with a thin layer of plasma polymerized allylamine (AA) or 2-methyl-2-oxazoline (POx) in a custom-built plasma reactor. **b** Gold nanoparticles were synthesized by reduction of hydrogen tetrachloroaurate (HAuCl_4_) with trisodium citrate. **c** Plasma-polymerized allylamine and 2-methyl-2-oxazoline coated surfaces were immersed in AuNPs solution for 24 h. **d** Surfaces were added to the wine and then removed after 3, 6 or 24 h, and the concentrations of H_2_S, EtSH and MeSH in the wine were measured before and after treatment.

The capacity to remove hydrogen sulfide (H_2_S), methanethiol, and ethanethiol from spiked model wine solutions was investigated using two different sizes of gold nanoparticles deposited on 2-methyl-2-oxazoline (POx) and allylamine (AA) underlayers. As shown in Supplementary Fig. 3, surfaces with 68 nm diameter gold nanoparticles immobilized on POx were the most effective. This could be attributed to the strong covalent binding of gold nanoparticles to the POx surface. In contrast, the binding of gold nanoparticles to AA surface is via an electrostatic bond, reversable when solution pH and/or ionic strength is changed.

Based on these results, the POx/68 nm AuNPs platform was selected to further investigate the effect of exposure time on the removal of sulfur compounds from a model wine for effective VSC management. As shown in Supplementary Fig. 3 the longest contact time was the most effective and applied in follow-up experiments.

The detection thresholds for the selected VSCs are as follows, H_2_S 1.1–1.6 ug/L, MeSH 1.8–3.1 ug/L, and EtSH 1.1 ug/L^7^.

The effectiveness of the nanoengineered surfaces in the removal of H_2_S, methanethiol, and ethanethiol was evaluated in real wines and compared to copper fining. The results are shown in Fig. 2 and Supplementary Fig. 4. The concentration of H_2_S decreased in all investigated white wines and most red wines. The treatment using nanoengineered surfaces was as effective as copper fining for white wines and even more effective than copper for red wines, indicating that the newly developed technology can be used as an alternative to copper fining. The concentration of methanethiol and ethanethiol also was decreased in all investigated white wines, and the treatment was more effective than copper fining in most cases. As for red wines, the results were less evident as both treatments gave no significant differences except for red wines #1 and #6, where treatment with the nanoengineered surfaces decreased the methanethiol concentration significantly. Furthermore, the interference of sulfur dioxide (SO_2_) with H_2_S removal by surfaces and their effect on ‘tropical’ sulfhydryls was also investigated. SO_2_ is used as a preservative in wine because of its anti-oxidative and anti-microbial properties.

**Fig. 2:**
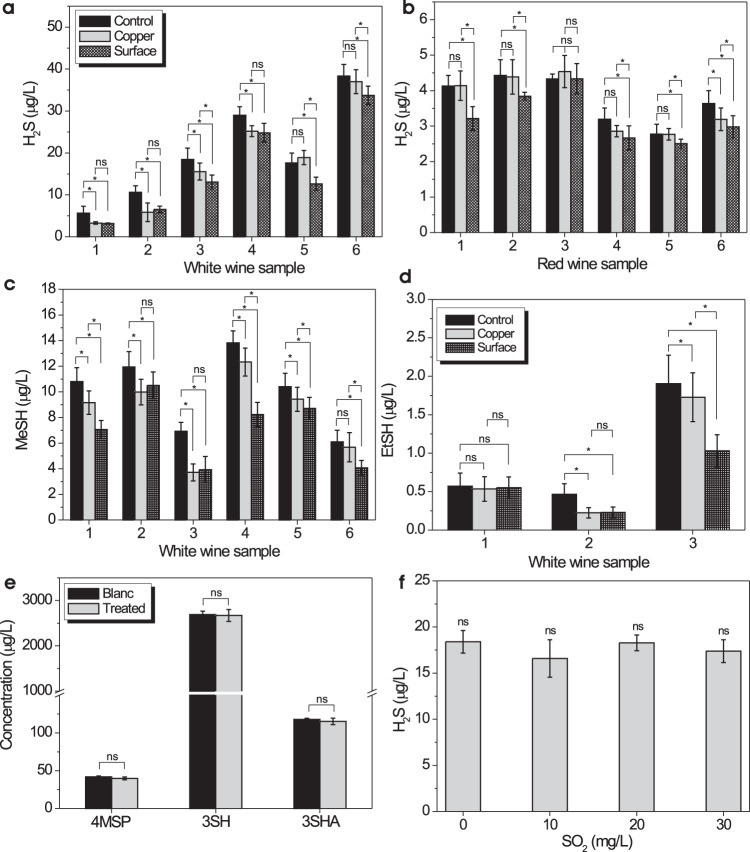
Concentration of volatile sulfur compounds in wines before and after treatment with nanoengineered surfaces that incorporate immobilized gold nanoparticles. H_2_S concentration **a** for white wines and **b** red wines, before and after treatment. Concentration of **c** methanethiol and **d** ethanethiol in white wines before and after treatment. **e** Concentration of tropical thiols 4MSP, 3SH and 3SHA in SAB before and after treatment. **f** Concentration of H_2_S in SAB as a function of SO2 addition to wine treated with surface. Each data point represents the average of at least three independently prepared samples. Means with a star are significantly different (*p* < 0.05) according to Student’s t test. Error bars indicate s.d.

As shown in Fig. 2f, SO_2_ does not interfere with the ability of the nanoengineered surfaces to remove H_2_S, and the nanoengineered surfaces do not remove tropical sulfhydryls. Both findings are significant. In contrast, when winemakers add copper to a finished wine to remove sulfides, they will also remove the volatile thiols^2^. While copper is quite effective at removing H_2_S and simple sulfhydryls, it is also known that copper has detrimental effects on wine aroma by decreasing more complex ‘tropical’ sulfhydryls. Considering that the compounds associated with tropical aroma are important for the stylistic expression of certain wine varieties with a clear ‘tropical fruit’ or ‘box hedge’ character, it is of utmost importance that these compounds remain in the wine. As shown in Fig. 2e, the concentration of 4-methyl-4-sulfanylpentan-2-one (4MSP, imparts box tree, passion fruit, and black currant aromas), 3-sulfanylhexan-1-ol (3SH, imparts grapefruit, passion fruit, gooseberry, and guava) and 3-sulfanylhexyl acetate (3SHA, imparts passion fruit, grapefruit, box tree, gooseberry, and guava aroma) were not altered after 24-hour treatment with nanoengineered surfaces. Copper and SO_2_ are common wine additives and can often be found in the wine at the same time. Whereas combined treatment of copper and SO_2_ can significantly increase H_2_S formation in wine samples^8^, this effect is not occurring in wines treated with nano nanoengineered surfaces as no increased H_2_S concentrations were measured by treating Sauvignon Blanc wine with 10, 20 or 30 mg/L SO_2_ (Fig. 1f).

We have developed and shown that nanoengineered surfaces can selectively remove unwanted ‘reductive’ aromas from the finished wine without altering a wine’s tropical fruit character. The ability of the nanoengineered surfaces to remove the ‘total’ fraction and the ‘free/non-metal bound’ fraction of unwanted sulfhydryl compounds was assessed in real wine. We found that a fraction of strongly bound sulfhydryls cannot be removed with neither the nano nanoengineered surfaces nor traditional copper fining. However, ‘free’ sulfhydryls were readily removed with the newly developed surfaces. The platforms were more effective in removing methanethiol than copper fining, with up to four times greater amounts of methanethiol removed when using the nanoengineered surface compared to using copper fining. Potentially, these nanoengineered surfaces could be adopted for common filtration apparatus, remediation application devices, new aerators and decanters, wine packaging material, and wine closures.

## Methods

### Materials

Sodium sulfide nonahydrate (98%), sodium thiomethoxide (95%), ethanethiol (99.7%), ethylmethyl sulfide (96%), potassium metabisulfite (98%) were obtained from Sigma-Aldrich (Castle Hill, NSW, Australia). Tartaric acid and sodium chloride was obtained from Merck (Frenchs Forest, NSW, Australia); absolute ethanol from Rowe Scientific (Lonsdale, SA, Australia); and copper (II) sulfate pentahydrate was obtained from Ajax Chemicals (Sydney, NSW, Australia). Water was obtained from a Milli-Q purification system (Millipore, North Ryde, NSW, Australia).

Allylamine (AA) (reagent grade, 98%) and 2-methyl-2-oxazoline (POx) (98%) were obtained from Sigma-Aldrich (Australia) and used as supplied. Microscope slides and 100-mesh stainless steel sheets were used as a substrate for plasma deposition.

### Analysis of volatile sulfur compounds

Volatile sulfur compounds were analyzed using gas chromatography with sulfur chemiluminescence detection (GC-SCD) as described in Siebert et al.^7^. Tropical sulfhydryls were measured using LCMS according to Capone et al.^9^.

### Spiking experiments

Model wine solutions were used to evaluate the effectiveness of the surfaces to remove VSCs, to determine the optimum treatment time to remove VSCs, as well as evaluate whether SO_2_ interfered with the ability of the smart surface to remove undesirable VSC from wine. Oxygen free model wine (<1 ppb oxygen) was prepared in an anaerobic hood by adding degassed ethanol (<1 ppb oxygen) to degassed MilliQ water (<1 ppb oxygen) that was previously pH adjusted to pH 3.6 using tartaric acid. The oxygen-free model wine (10 mL) was added to 22 mL amber vials inside an anaerobic hood (<1 ppb oxygen). Stock solutions of hydrogen sulfide, methanethiol, and ethanethiol were prepared inside an anaerobic hood using degassed MilliQ water and the stock solutions were added to the model wine to give final concentrations of approximately 25 μg/L for each VSC. The smart surfaces were then added to the vials containing model wine containing VSCs inside the anaerobic hood and the vials sealed with solid PTFE caps. To evaluate the different coating materials, the VSC analysis was carried out after 24 h. To determine the optimum treatment time, the VSC analysis was carried out after 3 h, 6 h, and 24 h.

To evaluate the effect of SO_2_ on VSC binding to the nanoengineered surface, the oxygen-free SO_2_ solutions were prepared inside an anaerobic hood using degassed Milli Q water and then added to the oxygen-free model wine to give final SO_2_ concentrations of 10, 20, and 30 mg/L. The smart surfaces were inserted into the oxygen-free model wine containing hydrogen sulfide and sulfur dioxide, the vials sealed using solid PTFE caps, and stored inside the anaerobic hood for 24 h. The treated wine samples were removed from the anaerobic hood after 24 h and VSC analysis performed.

Chardonnay, Sauvignon Blanc and Shiraz wines from the 2020 and 2021 vintages produced in South Australia were obtained from local wineries. The wines were pre-screened for VSC concentrations and wines with naturally high levels of hydrogen sulfide, methanethiol, and ethanethiol were selected for this trial. These wines were used to determine the effectiveness of the surface in removing VSCs naturally present in wine, to compare the effectiveness of the smart surface compared to copper fining, and to evaluate whether the smart surfaces remove desirable tropical sulfhydryls. To evaluate the effectiveness of the surface in removing VSCs naturally present in wine, the smart surfaces were placed inside 42 mL vials inside the anaerobic hood, 40 mL of each wine were added, and the vials were sealed with solid PTFE caps and stored in the anaerobic hood for 24 h.

To compare the effectiveness of VSC removal between copper fining and remediation using the smart surface, oxygen-free copper solutions were prepared inside an anaerobic hood using degassed MilliQ water and added to a subset of wine (40 mL) to give final concentrations of 0.1 mg/L copper. The vials were sealed with solid PTFE caps and stored in the anaerobic hood for 24 h.

To evaluate whether the smart surfaces remove desirable tropical sulfhydryls, the smart surfaces were placed inside 150 mL Schott bottles inside the anaerobic hood, wine (120 mL) containing natural concentrations of 4-MSP, 3-SH and 3-SHA were added, the vessels sealed with solid PTFE caps and stored in the anaerobic hood for 24 h.

All treated wines were removed from the anaerobic hood after 24 h and VSC analysis performed as described by Siebert et al. (2010). All samples were prepared in triplicate.

### Plasma polymerization

Allylamine (AA) (reagent grade, 98%) and 2-methyl-2-oxazoline (POx) (98%) were obtained from Sigma-Aldrich (Australia) and used as supplied. Microscope glass slides and 100-mesh stainless steel sheets were used as a substrate for plasma deposition. Plasma polymerization was carried out in a custom-built reactor equipped with a 13.56 MHz plasma generator^10^. Allylamine was deposited at a precursor pressure of 0.13 mbar, and 2-methyl-2-oxazoline at 0.08 mbar. The power used for deposition of both monomers was 40 W and 50 W, respectively. In both cases, the plasma deposition time was two minutes. Before deposition, all surfaces were cleaned by applying air plasma for 2 min at 50 W.

### Synthesis of gold nanoparticles (AuNPs)

Gold nanoparticles were synthesized by reducing hydrogen tetrachloroaurate (HAuCl_4_) with trisodium citrate. A 50 mL solution of 0.01% HAuCl4 was brought to boiling temperature with vigorous stirring. Under vigorous stirring, 1% water solution of trisodium citrate (TSC) was added. To achieve particle sizes of 38 and 68 nm in diameter, 0.5 mL and 0.3 mL of TSC were added, respectively. After adding trisodium citrate, the color of the solution changed from light yellow to wine red within minutes. The solution was kept for a further 20 min at boiling temperature and then cooled to room temperature^11^.

### Immobilization of gold nanoparticles

Plasma-polymerized allylamine and 2-methyl-2-oxazoline coated surfaces were immersed for 24 h in 38 and 68 nm AuNPs solution. Allylamine carries a positive charge when placed in an aqueous solution, while carboxylic acid groups functionalized AuNPs have a net negative charge. Immersion of AA-coated surfaces in AuNPs solution leads to strong electrostatic binding of the nanoparticles to the surface. After the gold nanoparticles binding, the surfaces were washed with water to remove loosely bound nanoparticles and dried in a vacuum. In the case of POx, these plasma polymer coatings are known to retain a population on intact oxazoline rings which bind covalently nanoparticles and other entities carrying COOH functionalities^12,13^.

### X-ray photoelectron spectroscopy (XPS)

XPS spectra were obtained using a Kratos Axis Ultra XPS spectrometer (Kratos Analytical Ltd, UK) with a monochromatic Al source and operated at 15 keV and 15 mA to get a survey spectrum from 0 eV to 1100 eV for all surface coatings. To compensate for surface charge effects, all the binding energies were referenced to the C1s neutral carbon peak at 285 eV. CasaXPS software was used for processing and curve fitting.

### Thickness measurements

The thickness of the deposited plasma polymers was determined using a variable angle ellipsometer (VASE, J. A. Woolam Co. USA). The experimental data were analyzed by WVASE32 (J. A. Woolam) software. The optical properties of the silicon wafer and the native oxide layer were taken from the software. A refractive index of 1.55^14^ was assumed for all plasma polymer layers.

### Contact angle

The contact angle was measured using the sessile drop method with a custom-made contact angle goniometer. A water droplet was placed onto the surface. Images of the droplet were taken with a horizontal digital microscope. The contact angles were determined by drawing the tangent near the edge of the droplet using the drop shape analysis software ImageJ with the DropSnake plugin. The experiments were conducted at room temperature in a clean room.

### Fourier transform infrared spectroscopy (FTIR)

IRTracer-100 FTIR spectrometer (Shimadzu) equipped with liquid nitrogen cooled MCT detector was used for all measurements. Measurements were performed using the Quest Single Reflection ATR Accessory (Specac), fitted with a diamond ATR crystal. In all cases, 128 scans at a resolution of 4 cm^−1^ were taken to obtain a satisfactory signal-to-noise ratio. The ATR effect and atmospheric contributions from carbon dioxide and water vapor were corrected by background performed on an empty ATR device.

### Scanning electron microscopy (SEM)

SEM was employed to determine the morphology and density of gold nanoparticles immobilized to the surface. An FEI Quanta 450 FEG-ESEM equipped with an EDAX Apollo X Energy Dispersive X-Ray (EDX) spectrometer was used for analysis. SEM images were analyzed using Image J software. For calculating number of nanoparticles per μm^2^, % surface coverage, and interparticle distance we have prepared three samples per nanoparticle size. These samples were analyzed by taking three images per sample.

### Statistical analysis

Data significance was assessed by Student’s *t* test. Data are presented as means ± (SD). *P* < 0.05 was considered statistically significant. All experiments were repeated at least three times. Figures were prepared using Origin 6.0 and CorelDRAW 11 software.

### Reporting summary

Further information on research design is available in the Nature Research Reporting Summary linked to this article.

## Supplementary information

Supplementary Information

Reporting Summary

## Data Availability

The authors declare that the data supporting the findings of this study are available within the paper and supplementary information files. The data also can be available on reasonable request from the corresponding author.
