# Supplementary material for: Surface nanoengineering technology for the removal of sulfur compounds associated with negative attributes in wines
Source: NPJ Sci Food. 2023 Feb 8;7:5. doi: 10.1038/s41538-023-00180-8 (PMC9905054; doi:10.1038/s41538-023-00180-8)
Supplement: Supplementary file 1 — Supplementary Information [file 41538_2023_180_MOESM1_ESM.pdf]

Supplementary Information

Surface nanoengineering technology for the removal of sulfur compounds associated with negative attributes in wines

Agnieszka M. Mierczynska-Vasilev[1]*, Allie C. Kulcsar[1], Panthihage Ruvini L. Dabare[2], Krasimir A. Vasilev[2], Marlize Z. Bekker[1]

[1] The Australian Wine Research Institute, Waite Precinct, Hartley Grove cnr Paratoo Road, Urrbrae (Adelaide) SA 5064, Australia

[2] College of Medicine and Public Health, Flinders University, Sturt Road, Bedford Park, SA 5042, Australia

* Corresponding author:

Agnieszka M. Mierczynska-Vasilev, agnieszka.mierczynska-vasilev@awri.com.au

We chose gold nanoparticles because they can be easily synthesized in a controlled manner and are chemically stable in the size range used in this study[1]. In addition, silver-based processing aids are typically not permitted to be used in winemaking. Furthermore, an advantage of using gold nanoparticles over, for example, silver nanoparticles is that the leaching behaviour of silver and gold are significantly different. Silver leaching occurs quite rapidly due to oxidation and dissolution of silver atoms at the surface, while the chemical stability of the gold nanoparticles above 10 nm in diameter prevents such processes from occurring[2].

We started with microscope glass slides as model substrates for a proof-of-concept demonstration. The surfaces were coated in a custom-built plasma reactor [3] with a thin layer of plasma polymerized allylamine (AA) or 2-methyl-2-oxazoline (POx). The

AA and POx contained a population of amine functional groups, which were positively charged in an aqueous medium under a pH of eight [4]. Colloidal gold nanoparticles of 38 and 68 nm in diameter were synthesized by the citrate reduction method [5]. Gold nanoparticles synthesised via this method are covered by unreacted citrate molecule and products of the reduction, and carry a negative surface charge[1]. Therefore, immobilization of gold nanoparticles on plasma-polymerized AA surfaces took place via electrostatic interaction. POx chemistry facilitated covalent reaction (covalent amide bonds) with carboxylic acid groups of sodium citrate.

Supplementary Figure 1 summarizes the physicochemical properties of allylamine and 2-methyl-2-oxazoline coatings deposited on glass slides. The thickness of the plasma polymers was in the range of 20 to 30 nm. Both coatings were hydrophilic. The IR and C1s high-resolution spectra revealing the complex chemistry of a typical plasma deposited POx and AA films are shown in Supplementary Figure 1. The elemental composition and atomic concentration of the chemical elements after immobilization of gold nanoparticles on those coatings are also given in Supplementary Figure 1. The gold atomic concentration was higher for the larger nanoparticles and was around 9 at. % on both POx and AA base layers.

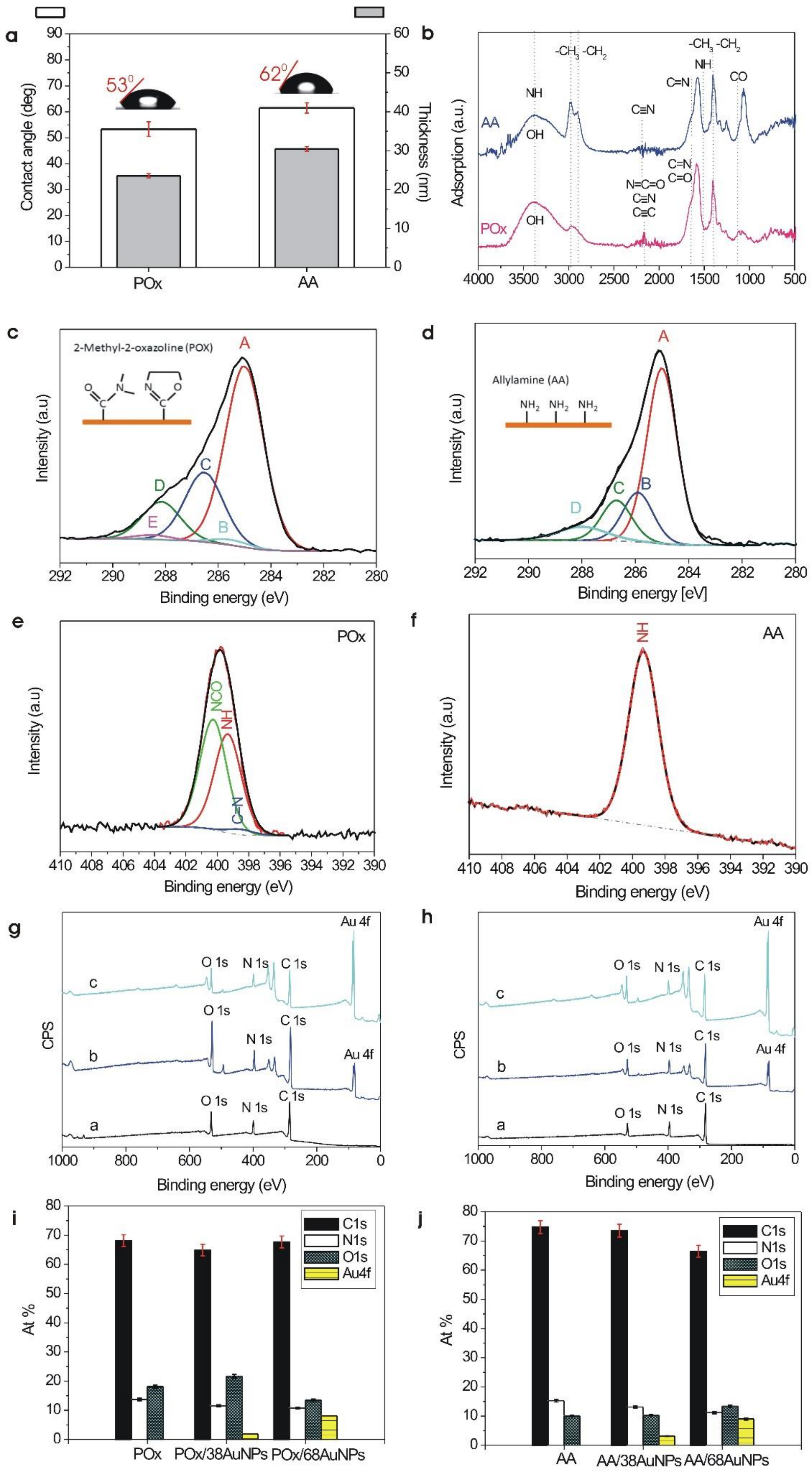
a
b
c
d
e
f
g
h
i
j
Contact angle (deg)
Thickness (nm)
53⁰
62⁰
POx
AA
Adsorption (a.u.)
-CH3 -CH2
NH
OH
C≡N
C=N
CO
N=C=O
C=O
C≡C
Intensity (a.u)
Binding energy (eV)
Binding energy [eV]
2-Methyl-2-oxazoline (POX)
Allylamine (AA)
NH2
NCO
NH
C=N
CPS
O 1s
N 1s
C 1s
Au 4f
At %
C1s
N1s
O1s
Au4f
POx/38AuNPs
POx/68AuNPs
AA/38AuNPs
AA/68AuNPs

Supplementary Figure 1. Physicochemical properties of allylamine and 2-methyl-2-oxazoline coatings.

a Plasma deposited 2-methyl-2-oxazoline and allylamine coatings thicknesses determined by Ellipsometry and advancing water contact angles. **b** FTIR spectra of 2-methyl-2-oxazoline (pink) and allylamine (blue) coatings deposited on KBr. **c** High-resolution C1s spectrum of 2-methyl-2-oxazoline coating. **d** High-resolution C1s spectrum of allylamine coating. **e** XPS high-resolution N1s spectrum of 2-methyl-2-oxazoline coating and **f** XPS high-resolution N1s spectrum of allylamine coating.

g XPS survey spectra showing the surface chemical composition of 2-methyl-2-oxazoline coated surface and surface modified with 38 and 68 nm Au nanoparticles

h XPS survey spectra showing the surface chemical composition of allylamine coated surface and surface modified with 38 and 68 nm Au nanoparticles. **i** Elemental composition of the allylamine coating before and after AuNPs immobilization. **j** Elemental composition of the 2-methyl-2-oxazoline coating before and after AuNPs immobilization. Errors bars indicate s.d.

Thickness measurements and contact angle

The thickness of the deposited plasma polymers was determined using an ellipsometer. Sample coatings' thicknesses are shown in Supplementary Figure 1a. For the plasma deposition conditions given in the Materials and Methods section, the thickness of the 2-methyl-2-oxazoline coating was 25 nm, whereas the thickness of the allylamine was 30 nm.

The wettability of investigated coatings was assessed by measuring the advancing contact angle of water on the 2-methyl-2-oxazoline and allylamine coatings. Both coatings were hydrophilic with water contact angles of 53° and 62°, as shown in

Supplementary Figure 1a. The measured contact angles align with previously published data [6]. Furthermore, they are evidence that the changes in surface chemical composition influence the wettability of the coatings and thus strongly correlate with the interfacial interactions.

FTIR analysis

Fourier transform infrared spectroscopy was used to identify the chemical functionalities present in the 2-methyl-2-oxazoline and allylamine coatings. The spectra of the investigated coatings are shown in Supplementary Figure 1b. A typical infrared spectrum of 2-methyl-2-oxazoline plasma coating is shown in Supplementary Figure 1b in pink. The coating contains many chemical functionalities, including C-O (1130 cm^{-1}) and C=N stretching (1650 cm^{-1}), both bonds present in the closed oxazoline ring. Since the C=N peak is quite broad, it likely overlaps with the C=O band, a function present in the open ring configuration of POx. In addition, the bands at ~2200 cm^{-1} indicate the presence of alkyne or isocyanate and nitrile functionality, which are specific to POx coatings.

The FTIR spectrum of allylamine is given in Supplementary Figure 1b in navy-blue. According to the spectrum, the basic features of the monomer allylamine are preserved. The symmetric/asymmetric stretching vibrations of $-CH_2$ and $-CH_3$ at 2980-2880 cm^{-1} and the deformation vibrations of these groups at 1456-1374 cm^{-1} are evidence of the radical chain-growth polymerization. The stretching vibrations of -NH at 3363 cm^{-1} and the deformation vibrations of amines at 1635 cm^{-1} indicate good retention of the amine groups. A new band of 2182 cm^{-1} associated with stretching vibrations of nitrile groups and the imine group and ethene group at about 1635 cm^{-1} suggests that the amino groups of the precursor were partially

transformed into amide, imine, or nitrile functional groups during the process of plasma deposition.

XPS analysis

In agreement with the precursor formula, the representative XPS survey spectrum of POx coating revealed that coating contains carbon (68%), oxygen (18%), and nitrogen (14%) (Supplementary Figure 1g(a)). Both the carbon and nitrogen environments are complex. The nitrogen peak in XPS high-resolution N1s spectrum for POx mainly has amine (399.2 eV) bonds and amide (400.1 eV), and imine functionality below the amine because of an increase in electron density due to conjunction (Supplementary Figure 1e). As presented in Supplementary Figure 1c, five distinct components were fitted in the high-resolution C1s spectrum, namely C-H/C-C (285 eV), COOH/R (289.7 eV with a β-shift peak at 285.9 eV), C-N/C-O, and C=O/C=N/NCO. The binding energies of these last two components were not set arbitrarily as slight differences are expected between the amide structure and the oxazoline ring.

The XPS survey scans were made of the coating prepared from allylamine (Supplementary Figure 1h(a)). Those deposits contained carbon (75%), nitrogen (15%) and oxygen (10%). The C1s spectrum of allylamine modified substrates is shown in Supplementary Figure 1d. The C1s core level spectrum of the coating was peak fitted for nitrogen-containing functionalities. The C1s peak accommodated four components: one at 285 eV, second at 285.9 eV, third at 286.7 eV, and fourth at 288 eV, which can be assigned to C−H or/and C−C, C−N, C=N, and CNO, respectively. The N1s spectrum showed a pronounced nitrogen peak, which is characteristic of allylamine plasma polymers, as shown in Supplementary Figure 1f.

Supplementary Figure 1g shows XPS survey spectra of POx coating and POx coating with immobilized 38 (Supplementary Figure 1g(b)) and 68 nm (Supplementary Figure 1g(c)) Au nanoparticles. Supplementary Figure 1h shows XPS survey spectra of allylamine coating and allylamine coating with immobilized 38 (Supplementary Figure 1h(b)) and 68 nm (Supplementary Figure 1h(c)) Au nanoparticles. The survey spectra of coatings without AuNPs (Supplementary Figures 1g(a) and 1h(a) show the presence of carbon (C1s), oxygen (O1s), and nitrogen (N1s) only which is consistent with the chemical composition of materials. After immobilizing gold nanoparticles of varying sizes to those surfaces, extra peaks appear corresponding to gold (Au) (Supplementary Figures 1g(b) and 1g(c) and Supplementary Figures 1h(b) and 1h(c)). The presence of the Au peaks shows that the coatings were successfully modified with gold nanoparticles. In addition, the 68 nm AuNPs immobilised on plasma-modified surfaces exhibit a higher amount of gold than the 38 nm AuNPs as shown in Supplementary Figures 1i and 1j. For the allylamine plasma-modified surface, the atomic % of gold increased from 3 to 9 at.% when the particle size of immobilized particles increased from 38 to 68 nm. For POx modified surface, the increase of gold at.% was 2.5 and 8.5 at.% for 38 and 68 nm AuNPs, respectively.

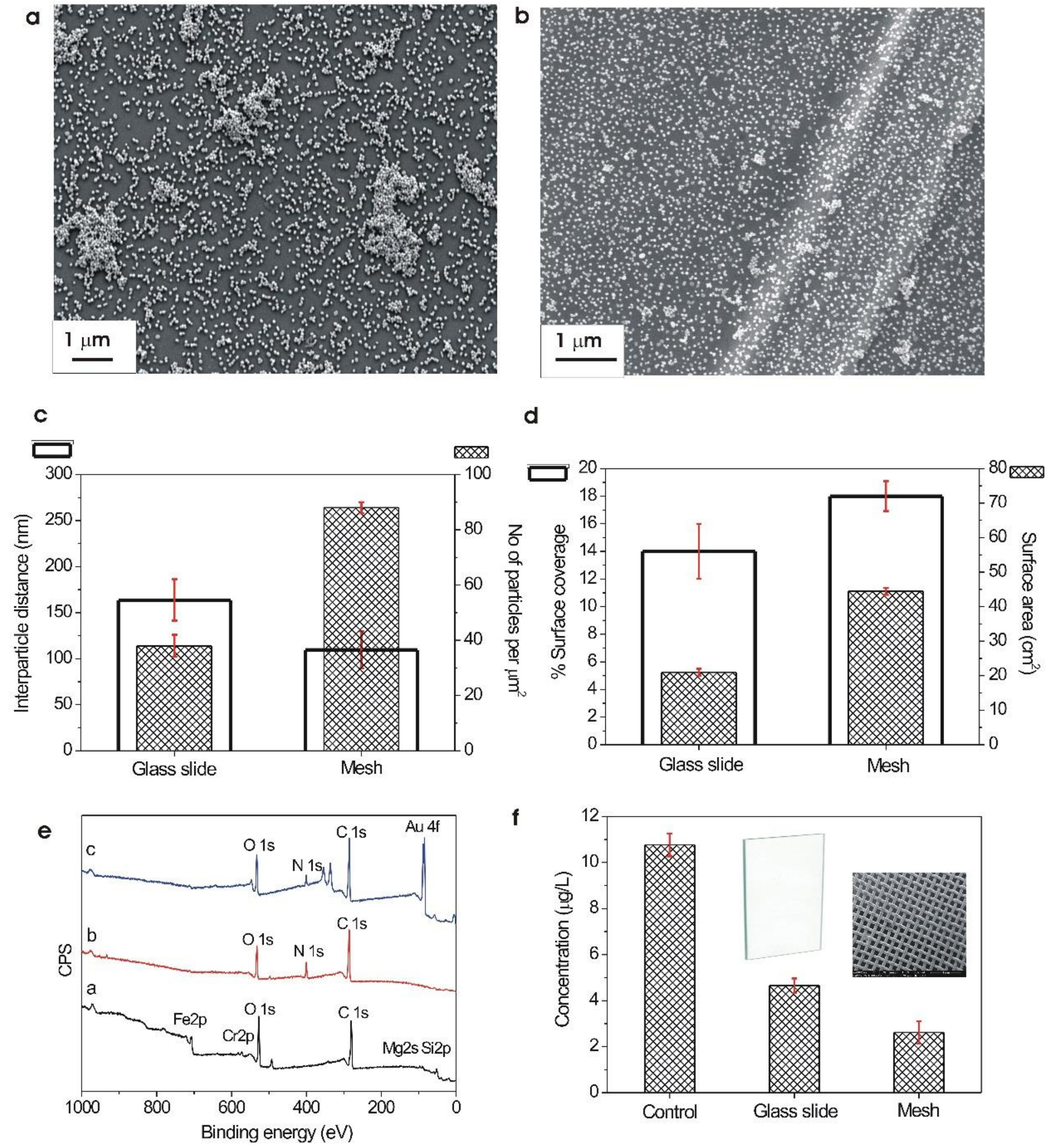

Supplementary Figure 2. Physicochemical properties of coatings deposited on glass and mesh surfaces. **a** Scanning electron microscopy (SEM) image of 68 nm gold nanoparticles on POx was deposited on a glass slide. **b** SEM image of 68 nm gold nanoparticles on POx deposited on a mesh surface. **c** Interparticle distance and number of particles calculated from SEM images by Image J software. **d** % surface coverage with AuNPs and surface area of a glass slide and mesh surface. **e** XPS survey spectra showing the surface chemical composition of bare mesh surface (a),

mesh surfaces coated with POx (b), and mesh surfaces coated with POx and modified with 68 nm Au nanoparticles (c). **f** Concentration of hydrogen sulfide in white wine after treatment with 68 nm AuNPs/POx deposited on a glass slide and mesh surface. Errors bars indicate s.d.

In plasma deposition, film growth occurs independently of the substrate material used [7]. For this reason, we could directly transfer the surface modification technology from model glass substrates to stainless steel mesh sheets. As presented in Supplementary Figure 2, the mesh was more effective in removing H_2S from wine. The reason for that was the significant increase (more than double) in the substrate available surface area. It was also possible to coat both sides of the mesh sheet, which increased the number of gold nanoparticles attached to the surface. In addition, from practical perspective, using an adsorbent mesh is much more convenient, especially in industrial applications as this surface can be applied to in-line filtration systems. Surface analysis using scanning electron microscopy (SEM) were carried out in order to derive a comprehensive picture of the nanoengineered surfaces in terms of the number of nanoparticles per mm^2, interparticle distance, surface coverage, and surface area. The number of nanoparticles per mm^2 calculated from the SEM images using the Image J software is shown in Supplementary Figure 2c. Immobilisation of AuNPs on the glass slide resulted in 40 particles per mm^2, while 88 particles per mm^2 was found on the mesh surface. The distance between the particles was 160 nm and 100 nm for the glass surface and the mesh surface, respectively. The surface coverage of the nanoparticles (Supplementary Figure 2d) was 14 % and 18 % for nanoparticles with a diameter of 68 nm immobilised on the glass and mesh surface, respectively.

The SEM images were further used to measure the dimensions of the nanoparticles, which were used to calculate the volume and mass of the nanoparticles. Assuming 19.32 g/cm^3 as the density of gold nanoparticles, the mass of a 68 nm nanoparticle is 25 femtogram. Taking this into account, the mass of 68 nm nanoparticles is 2.2 picogram per 1 mm^2 (0.22 mg/cm^2).

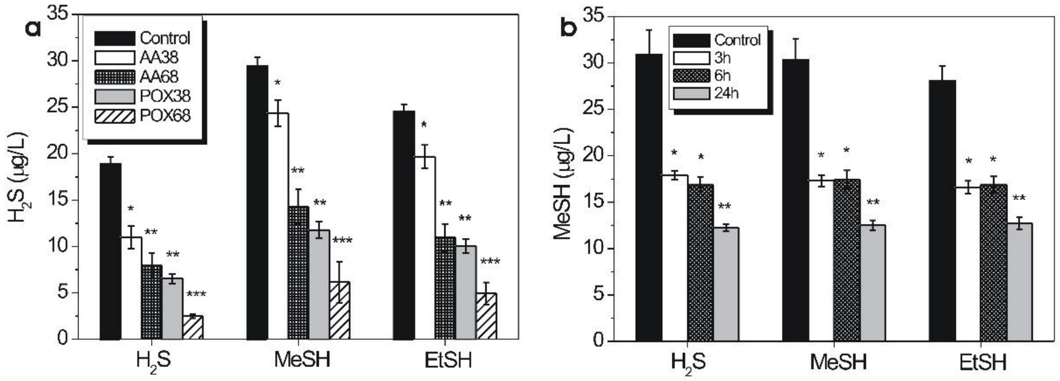

Supplementary Figure 3. Concentration of volatile sulfur compounds in spiked model wine solutions. **a** Concentration of hydrogen sulfide (H_2S), methanethiol (MeSH), and ethanethiol (EtSH) in spiked model wine solution after treatment with 38 and 68 nm gold nanoparticles deposited on 2-methyl-2-oxazoline and allylamine coatings. **b** Concentration of hydrogen sulfide (H_2S), methanethiol (MeSH), and ethanethiol (EtSH) in spiked model wine solution after three, six, and 24-hour treatment with 68 nm gold nanoparticles deposited on 2-methyl-2-oxazoline surface. Errors bars indicate s.d.

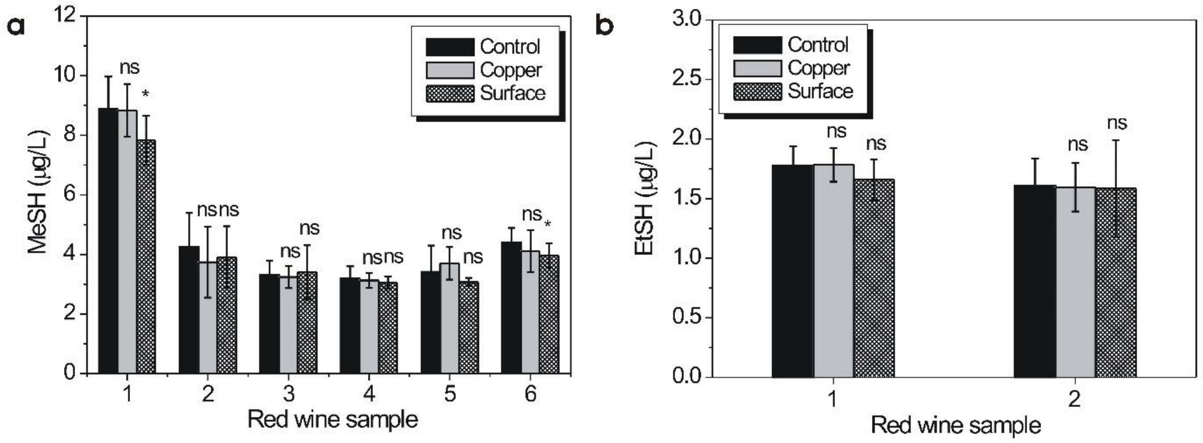

Supplementary Figure 4. Concentration of volatile sulfur compounds in red wines. The concentration of **a** methanethiol and **b** ethanethiol in red wine samples before and after treatment with copper and surfaces. Errors bars indicate s.d.

References

1 Zhu, T., Vasilev, K., Kreiter, M., Mittler, S. & Knoll, W. Surface modification of citrate-reduced colloidal gold nanoparticles with 2-mercaptosuccinic acid. *Langmuir* **19**, 9518-9525 (2003).

2 Jeffrey, M. I. Kinetic aspects of gold and silver leaching in ammonia-thiosulfate solutions. *Hydrometallurgy* **60**, 7-16 (2001).

3 Vasilev, K. *et al.* Early stages of growth of plasma polymer coatings deposited from nitrogen-and oxygen-containing monomers. *Plasma Processes and Polymers* **7**, 824-835 (2010).

4 Mierczynska, A. *et al.* pH-tunable gradients of wettability and surface potential. *Soft Matter* **8**, 8399-8404 (2012).

5 Turkevich, J., Stevenson, P. C. & Hillier, J. A Study of the Nucleation and Growth Processes in the Synthesis of Colloidal Gold. *Discuss Faraday Soc*, 55-75 (1951).

6 Mierczynska-Vasilev, A. & Smith, P. A. Adsorption of Wine Constituents on Functionalized Surfaces. *Molecules* **21**, 1-14 (2016).

7 Vasilev, K., Michelmore, A., Griesser, H. J. & Short, R. D. Substrate influence on the initial growth phase of plasma-deposited polymer films. *Chemical Communications*, 3600-3602 (2009).
